# Relationship between acute stress and sleep disorder in grass-root military personnel: mediating effect of social support

**DOI:** 10.1186/2054-9369-1-3

**Published:** 2014-04-15

**Authors:** Qi-jun Zhang, Qiao-li Zhang, Xin-yang Sun, Li-yi Zhang, Si-yuan Zhang, Gao-feng Yao, Chun-xia Chen, Ling-ming Kong

**Affiliations:** Department of Psychology, 92919 Military Hospital, Jiangbei District, Ningbo, Zhejiang 315000 PR China; Postgraduate School, Xuzhou Medical College, Xuzhou, Jiangsu 221002 PR China; Department of Psychology, The Second Military Medical University, Shanghai, 200433 PR China; Prevention and Treatment Centre for Psychological Diseases of PLA, the PLA 102nd Hospital, Changzhou, 213003 PR China; Public Health Division of Joint Logistics Department, Nanjing Military Area Command, Nanjing, 210000 PR China

**Keywords:** Grass-root military personnel, Acute stress, Social support, Sleep disorder, Structural equation model

## Abstract

**Background:**

Sleep disorder induced by acute stress has always been an important topic for study among the general population. However, the mediating effect of social support between acute stress and sleep disorder has rarely been reported before.

**Methods:**

A total of 2,411 grass-root military personnel were randomly selected by cluster sampling, and administered the Chinese Military Personnel Sleep Disorder Scale, Military Acute Stress Scale and Social Support Rating Scale.

**Results:**

The total score of acute stress scale was positively correlated with the total score and factor scores of sleep disorder scale (*r* = 0.209 ~ 0.465, *P* < 0.01); The total score of social support scale was positively correlated with the total score of acute stress scale and the total score and factor scores of sleep disorder scale (*r* = 0.356 ~ 0.537, *P* < 0.01). The analysis of mediating effects showed that lack of social support partially mediated between acute stress and the factors of sleep disorder. The analysis of structural equation model showed that acute stress not only had a direct effect on sleep disorder (the path coefficient was 0.29, *P* = 0.000), but also on lack of social support (the path coefficient was 0.39, *P* = 0.000); lack of social support had a direct effect on sleep disorder (the path coefficient was 0.48, *P* = 0.000).

**Conclusions:**

Acute stress and lack of social support are two significant factors of sleep disorder in grass-root military personnel. Well-established social support could alleviate sleep disorder induced by acute stress. Lack of social support was a partial mediator between acute stress and sleep disorder.

## Background

Stress has been a significant predisposing cause for various major fatal diseases. Military stress is defined as a special emotional state under extraordinary military circumstances, which might physiologically and psychologically exert negative influences upon individuals [1]. Acute stress could potentially give rise to anxiety, irritability, sleep disorders in particular, and other symptoms as acute emotional reaction, and might compromise individuals’ functions in social, occupational and other significant fields [2]. Sleep quality has aroused extensive concerns, since it plays a significant role in the normality and quality of a wide range of psychological and physical functioning at the awakening time. Sleep disorder might severely impair life quality and downgrade working efficiency [3]. Cognitive stress theory has it that stress reactivity can’t be defined as simple stimulation-induced reaction, but is determined by multiple mediating factors, such as social support, individual cognitive evaluation and others [4]. Based upon the study by Wenyu et al., social support of the grass-root military personnel could impair their sleep quality [5]. Generally speaking, social support, acute stress and sleep disorder are closely linked with each other. But there’s barely any research combined these three factors of the grass-root military personnel. This study, taking grass-root military personnel as study subjects, investigated the relationship between these three factors by mediating effect analysis and pathway analysis, in an effort to provide references for improving sleep quality in grass-root military personnel.

## Methods

### Measuring instruments

Social Support Rating Scale [6]: there are altogether18 items, covering three dimensions, namely subjective support, objective support and support utility rate, plus a lying factor. It adopted three levels scoring, with never scoring 0, sometimes scoring 1 and always scoring 2. Higher scores indicated lower social support. All coefficients have been verified as follows: the correlation coefficient between factors ranged from 0.48 to 0.59 (*P* < 0.01), and the correlation coefficient between factors and the general scale ranged from 0.72 to 0.82 (*P* < 0.01); the test-retest coefficient of the general scale and factors ranged from 0.62 to 0.80 (*P* < 0.01); Cronbach’α coefficients ranged from 0.62 to 0.87; split half coefficients ranged from 0.55 to 0.83. These verification results have demonstrated that this scale had excellent reliability and validity, meeting psychological assessment criteria.

Chinese Military Personnel Sleep Disorder Scale [7]: there are altogether 29 items, covering five dimensions, namely daily functioning, insomnia, hypersomnia, motile abnormal sleep and immotile abnormal sleep. It adopted four levels scoring, with never scoring 1, occasionally scoring 2, often scoring 3 and always scoring 4. Higher scores indicted more serious sleep disorder. All coefficients were verified as follows: the correlation coefficient between factors ranged from 0.30 to 0.50 (*P* < 0.01), and the correlation coefficient between factors and the general scale ranged from 0.40 to 0.83 (*P* < 0.01); the test-retest coefficient of the general scale and factors ranged from 0.62 to 0.88 (*P* < 0.01); Cronbach’α coefficients ranged from 0.35 to 0.67; split half coefficients ranged from 0.59 to 0.85. These verification results have demonstrated that this scale had excellent reliability and validity, meeting the psychological assessment criteria.

Military Acute Stress Scale [8]: there are altogether 42 items, covering nine dimensions, namely respiratory system, nervous system, cardiovascular system, skeletal system, digestive system, urogenital system, emotion, language and behavior, sleep, plus a lying factor. It adopted two levels scoring, with yes scoring 1 and no scoring 2. Higher scores indicted more serious stress-related symptoms. All coefficients were verified as follows: the correlation coefficient between factors ranged from 0.28 to 0.57 (*P* < 0.01), and the correlation coefficient between factors and the general scale ranged from 0.70 to 0.85 (*P* < 0.01); the test-retest coefficient of the general scale and factors ranged from 0.38 to 0.91 (*P* < 0.01); Cronbach’α coefficients ranged from 0.61 to 0.93; split half coefficients ranged from 0.47 to 0.86. These verification results have demonstrated that this scale had excellent reliability and validity, meeting the psychological assessment criteria.

### Testing procedure

All participants were divided into groups of about 30 to 50 individuals and were group-tested by automatic testing device. One research fellow made the leading remarks before the procedure, and three research fellows monitored the procedure, which lasted about half an hour. All participants were given the informed content before testing.

This study was approved by the Human Research Medical Ethics Committee at No. 102 Hospital of PLA. Informed consents were obtained from all participants. Details regarding the study methods have been reported previously.

### Quality control

All participants were screened for histories of psychological diseases, organic diseases and drug dependence. They were not requested to fill out the name of the responders to dispel misgivings. All questionnaires which were continuously, randomly or arbitrarily filled, or whose lying score was higher than the average plus 1.96 standard error, were excluded. All 2,490 pieces were recollected, among which 79 were excluded based upon standards mentioned above, making questionnaire validity rate 96.8%.

### Statistical analysis

Pearson correlation analysis and stratified regression analysis were performed in the platform of SPSS version 17.0. The stratified regression analysis was carried out as follows. Based upon the study by Zhonglin et al. [9], regression analysis was carried out to verify the mediating effect of social support between acute stress and sleep disorder. To begin with, acute stress, social support and sleep disorder were centralized processed (generating new variables by original variables subtracting its average). Step 1, to verify the regression coefficient by taking sleep disorder total score as dependent variable, and taking acute stress as independent variable; Step 2, to verify the regression coefficient by taking social support total score as dependent variable, and taking acute stress as independent variable; Step 3, to verify the regression coefficient by taking sleep disorder total score as dependent variable, and taking social support and acute stress as independent variables. If significant results were found in the first two steps, and no significant result was found in Step 3, then full mediating effect would be established; while if all three steps produce no significant results, then partial mediating effect would be established. The structural equation model was built in the platform of AMOS 7.0. It was carried out as follows. Structural equation model was constructed by taking acute stress as explicit variable for stress measurement, taking the three factors, including subjective support, objective support and support utility rate, for social support measurement, and taking the five factors, namely daily function, insomnia, hypersomnia, motile abnormal sleep and immotile abnormal sleep, for sleep disorder measurement. *P* < 0.01 was considered as statistically significant.

## Results

### Demographic variables by all the participants

Table 1 shows that the demographic information of all the participants.

**Table 1:** **Demographic variables by all the participants**

Military ranks		Military service duration			Age			Educational levels		
Soldier	Officer	Range	Mean	Median	Range	Mean	Median	Junior high	Senior high or secondary	College and above
2,404 (96.5%)	86 (3.6%)	1-18	3.24	3.06	17-36	20.93	3.58	456 (18.3%)	1954 (78.5%)	80 (3.2%)

### Correlation analysis of social support, sleep disorder and acute stress

Table 2 shows that the total score of social support scale was positively correlated with the total score and factor scores of sleep disorder (*P* < 0.01). It also shows the total score of acute stress scale was positively correlated with social support (*P* < 0.01); the total score of acute stress scale was positively correlated with the total score and factor scores of sleep disorder (*P* < 0.01).

**Table 2:** **Correlation analysis of relationship between social support, acute stress and sleep disorder (** ***r*** **)**

Variable	ST	AT	S1	S2	S3	S4	S5
AT	0.366^(1)^						
S1	0.537^(1)^	0.458^(1)^					
S2	0.509^(1)^	0.432^(1)^	0.893^(1)^				
S3	0.463^(1)^	0.465^(1)^	0.898^(1)^	0.739^(1)^			
S4	0.415^(1)^	0.362^(1)^	0.795^(1)^	0.681^(1)^	0.646^(1)^		
S5	0.405^(1)^	0.270^(1)^	0.753^(1)^	0.586^(1)^	0.578^(1)^	0.450^(1)^	
S6	0.356^(1)^	0.209^(1)^	0.652^(1)^	0.494^(1)^	0.419^(1)^	0.396^(1)^	0.638^(1)^

Significance levels of correlation coefficients between the social support and sleep disorder and acute stress: ^(1)^ *P* < 0.05. AT. Grass-root military personnel’s acute stress; ST. Grass-root military personnel’s social support; S1-S6. Factors of Grass-root military personnel’s sleep disorder, i.e. total scores, daytime dysfunction, insomnia, lethargy, motile abnormal sleep, immotilea abnormal sleep.

### Mediating effect analysis

Based upon the study by Zhonglin et al. [9], Table 3 shows that lack of social support partially mediated between acute stress and the factors of sleep disorder.

**Table 3:** **Stratified regression analysis of sleep disorder, acute stress and social support(** ***α***_**in**_ **= 0.05,** ***α***_**out**_ **= 0.10)**

Procedure	Dependent variable	Variable	***R*** ^***2***^	ΔR^2^	***F***	***B***	***β***
1	Sleep disorder	Acute stress	0.210	0.210	640.928^(1)^	0.696^(1)^	0.458^(1)^
2	Social support	Acute stress	0.134	0.134	373.049^(1)^	0.309^(1)^	0.366^(1)^
3	Sleep disorder	Social support	0.367	0.367	698.396^(1)^	0.766^(1)^	0.426^(1)^
		Acute stress				0.459^(1)^	0.303^(1)^

Significance levels of hierarchical regression analysis: ^(1)^ *P* < 0.01. *β*. Standardized regression analysis coefficient.

### Pathway analysis

**Figure 1:**
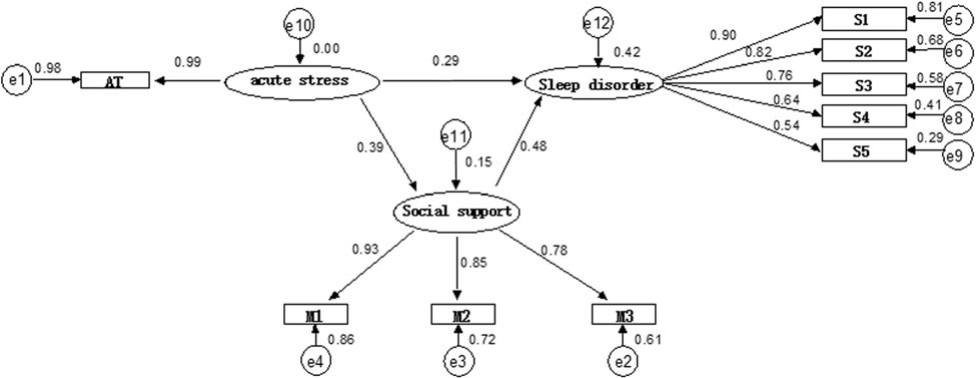
**Path model of grass-root military personnel’s acute stress, social support and sleep disorder.** AT. Grass-root military personnel’s acute stress; S1-S5. Factors of grass-root military personnel’s sleep disorder, i.e. daytime dysfunction, insomnia, lethargy, motile abnormal sleep, immotile abnormal sleep; M1-M3. Factors of grass-root military personnel’s social support, i.e. subject support, object support, the use of social support.

Maximum likelihood method was employed to calculate the major fitting indexes as follows: *x*^*2*^/*df* = 30.963 (*P* = 0.000), GFI = 0.931, AGFI = 0.877, CFI = 0.938, RMSEA = 0.112. This model was modified by assuming covariation between e1 and e6, e3 and e8, e6 and e8, e8 and e9, and better fitting indexes were generated as follows:*x*^2^/*df* = 7.596 (*P* = 0.000), GFI = 0.985, AGFI = 0.968, CFI = 0.989, RMSEA = 0.042 (see Figure 1). Based upon the study by Minglong [10], if AGFI > 0.900 and RMSEA < 0.05, the model fitting is good. So the model fitting of this study could be considered to be good.

## Discussion

The correlation analysis in this study demonstrated that acute stress and social support were positively correlated with sleep disorder and its factors, which might suggest that acute stress and lack of social support exert impact upon sleep quality, according well with other studies. Spoor-maker et al. concluded that sleep disorder constituted a prominent issue in acute stress, and more serious consequences could be brought about by acute stress events plus sleep disorder [11, 12]. Both Schoenfeld and Kobayashi investigated sleep quality in stressed individuals and found that acute stress would impair sleep quality and even engender sleep disorder [13, 14]. Hall et al. found that acute stress reactivity was significantly related to the down-regulation of parasympathetic nerves during non-rapid eye movement sleep (NREM sleep) and rapid eye movement sleep (REM sleep), and the heart rate abnormalities induced by acute stress could also impair sleep quality [15]. Krakow et al. has verified that treatment targeting sleep disorder could alleviate stress symptoms in over 50% patients [16], and another study by Brummett et al. showed that social support system also had indirect bearing on sleep quality [17]. Under military circumstances, rigorous and overloaded training might give rise to mental stress and over-fatigue and other stress responses; on the other hand, strained interpersonal relationship, family traumatic events and lack of social support could also induce stress responses [18]. All of these studies suggest that less acute stress and better social support system would greatly reduce sleep disorders and improve significantly sleep quality. However, another study by Hellhammer et al. proved that neuroendocrine response mechanism induced by acute stress played a positive role in improving sleep quality [19], which was inconsistent with our study. The reasons of the inconsistent might be that, the kinds of the participants were different, then the acute stress could be different either.

The results of stratified regression analysis showed that social support partially mediated between acute stress and sleep disorder, which was further confirmed by structural equation model construction. Both results verified that acute stress could directly lead to sleep disorder, and also could indirectly lead to sleep disorder through the mediating effect of social support. This conclusion is consistent with other previous studies. Yu et al. found that social support mediated between stressful life events and psychological symptoms [20]. Another study by Zhu et al. verified that extensive social support system could serve as cushion for effects of stressful life events upon emotion, thus preserving mental health [21]. Multiple studies have suggested that social support, defined as perception of external support available, served as an important variable in stress responses, and played a significant role in alleviating stress response, preserving mental health and improving sleep quality [22–24]. Based upon this study, extensive social support could improve sleep quality by reducing acute stress in grass-root military personnel.

## Conclusions

This study has demonstrated through mediating effect analysis and structural equation model construction that social support partially mediated between acute stress and sleep disorder, and well-established social support system and less acute stress could greatly improve sleep quality. This study is of great significance in providing references for improving sleep quality in grass-root military personnel and enhancing military combating capacity.
